# An incidental discovery of a silent tracheal bronchus during flexible bronchoscopy: a case report and anesthetic perspective

**DOI:** 10.3389/fped.2025.1718566

**Published:** 2026-01-06

**Authors:** Chaohui Zou, Pinyi Lv, Xu Li, Qianwen Yang, Lili Liu

**Affiliations:** 1 Department of Anesthesiology, Yan'an Hospital Affiliated to Kunming Medical University, Kunming, Yunnan, China; 2 Anesthesiology Operating Center, Yan'an Hospital Affiliated to Kunming Medical University, Kunming, Yunnan, China

**Keywords:** airway anomaly, case report, diagnostic bronchoscopy, incidental finding, pediatric anesthesia, tracheal bronchus

## Abstract

**Background:**

A tracheal bronchus is a rare congenital anomaly of the tracheobronchial tree. Although often asymptomatic, its presence carries profound implications for airway management, particularly in anesthesiology. Unrecognized cases can lead to life-threatening complications such as ventilation failure and lobar collapse.

**Case report:**

We report the case of a 5-year-old boy presenting with clinical and radiological features of left lower lobe bronchopneumonia. During a diagnostic flexible bronchoscopy performed under intravenous sedation-analgesia with preserved spontaneous ventilation, an anomalous right tracheal bronchus was discovered incidentally. This variant was entirely asymptomatic and contralateral to the infectious focus. The procedure was completed uneventfully, and the patient was discharged following successful medical management of *Mycoplasma pneumoniae* infection.

**Conclusion:**

This case highlights two critical aspects from an anesthetic perspective. First, it underscores the pivotal role of the anesthesiologist as a vigilant diagnostician in identifying clinically silent but high-stakes airway anomalies. Second, it demonstrates the utility of non-intubated sedation techniques in facilitating an unobstructed anatomical examination of the native airway. Proactive identification and documentation of such variants are essential for safeguarding future patient care.

## 1 Introduction

The tracheal bronchus (TB), first described in the 18th century, is a congenital aberration wherein a right upper lobe bronchus has its origin in the trachea rather than at the carina, typically within 2 cm proximal to the main carina (1, 2). Its reported prevalence ranges from 0.9% to 3% in children, with a strong right-sided predilection (3–5). Right upper lobe bronchial anomalies are seven times more common than left-sided ones, with a prevalence rate of 0.1%–2% on bronchographic examinations (2, 6). Recent studies utilizing advanced imaging and systematic bronchoscopic evaluation continue to affirm this prevalence and highlight that the majority of these anomalies remain clinically silent, often representing incidental findings (4, 7, 8). Although a TB can be associated with recurrent pulmonary infections, chronic atelectasis, or stridor due to impaired drainage or stenosis, it is estimated that the majority of cases are asymptomatic, and a significant proportion of individuals remain entirely asymptomatic throughout life; hence, they are often discovered incidentally during imaging or bronchoscopic procedures (6, 9).

**Figure 1:**
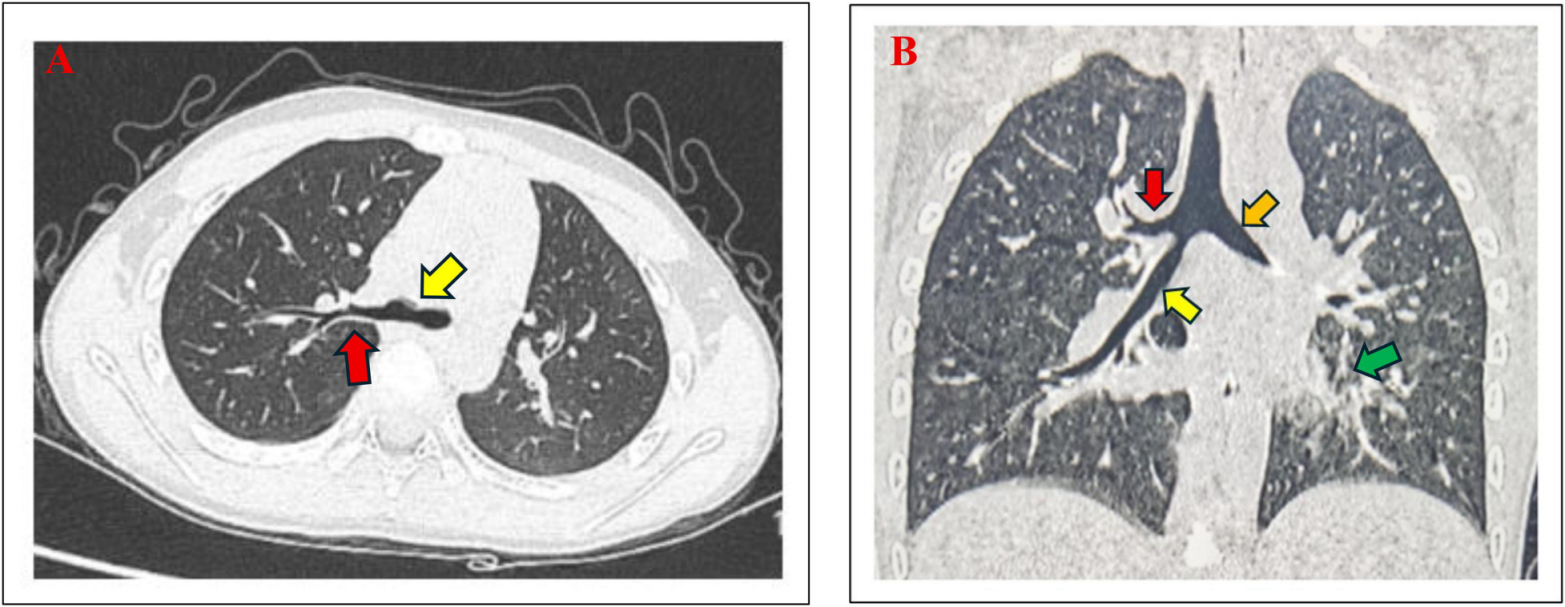
**(A,B)** Chest computed tomography (CT) findings. **(A)** Axial view (lung window) showed revealing tracheal bronchus (red arrow) over the right-sided distal tracheal (yellow arrow) above the carina. The right upper lobe bronchus was absent at its typical branching site. **(B)** Coronal view demonstrated the tracheal bronchus (red arrow) arising superior to the carina, along with the right main bronchus (yellow arrow) and left main bronchus (orange arrow). Patchy consolidation and mucus impaction were evident in the left lower lobe (green arrow).

From an anesthesiological standpoint, the TB is a recognized, although rare, component of the “difficult airway” algorithm. Failure to recognize it during endotracheal intubation can result in occlusion of the aberrant orifice by the endotracheal tube (ETT) cuff, leading to intraoperative hypoxemia and postoperative atelectasis of the supplied lung segment (10, 11). Furthermore, it presents a formidable challenge for achieving one-lung ventilation during thoracic surgery (6, 12). This case illustrates how an anesthesiologist's vigilance during routine bronchoscopy for pneumonia led to the diagnosis of an asymptomatic TB, thereby mitigating future anesthetic risks.

## 2 Case presentation

### 2.1 Patient information

A 5-year-old boy (height: 108 cm, weight: 17 kg) with no significant past medical history was admitted to the pediatrics department. The admission was prompted by persistent high fever (peak temperature 39.5°C) and clinical concerns regarding potential lobar consolidation requiring intravenous antibiotic therapy, following unsuccessful outpatient management with oral amoxicillin–clavulanate for 3 days. His vaccination status was complete and up to date.

### 2.2 Clinical findings and diagnostic assessment

On admission, his vital signs were as follows: temperature 38.9°C, heart rate 132 beats per minute, respiratory rate 28 breaths per minute, and oxygen saturation 96% on room air. Auscultation revealed diminished breath sounds accompanied by late-inspiratory coarse crackles over the left lower lung field, with an otherwise unremarkable physical examination. Laboratory investigations demonstrated leukocytosis (white blood cell count 11.5 × 10⁹/L) with neutrophilia (68%) and an elevated C-reactive protein level (12 mg/L, reference value < 8 mg/L).

The initial differential diagnosis for community-acquired pneumonia in this child encompassed typical bacterial pathogens (e.g., *Streptococcus pneumoniae*), atypical bacteria (e.g., *Mycoplasma pneumoniae*), and viral agents. The absence of a rapid response to broad-spectrum cephalosporin antibiotics increased our suspicion for an atypical pathogen. Furthermore, due to persistent fever and clinical deterioration after 48 h of intravenous ceftriaxone, chest computed tomography (CT) imaging was performed to raise the possibility of a tracheal bronchus. This incidental finding introduced a new anatomical consideration, prompting the decision to perform flexible bronchoscopy. Bronchoscopy was performed to definitively confirm the CT-suspected tracheal bronchus and rule out any associated obstructive complication that might be contributing to the clinical picture.

### 2.3 Therapeutic intervention

Because of a suboptimal clinical response to initial empiric antibiotic therapy, the patient's diagnostic and therapeutic management was progressively escalated. The key decisions and their timing are summarized in the clinical timeline (Table 1). This evolving course ultimately led to the decision to perform a diagnostic flexible bronchoscopy on hospital Day 4 to better characterize the pathology and identify the causative organism.

**Table 1:** Clinical timeline and management decisions.

Time point (day)	Key events and interventions	Clinical rationale and findings
Day 1	- Hospital admission. - Prior oral amoxicillin–clavulanate for 3 days. - Initiation of empiric intravenous antibiotics (*Ceftriaxone*)	- Persistent high fever and clinical signs of pneumonia necessitating inpatient care and IV therapy.
Day 3	- Persistent fever and lobar collapse on repeat chest X-ray. - Performance of chest computed tomography (CT).	- Persistent fever after 48 h of IV antibiotics. - To evaluate the extent of consolidation, exclude complications (e.g., abscess, effusion), and investigate poor clinical response. - *CT finding*: Confirmed left lower lobe consolidation and raised suspicion of a tracheal bronchus (Figures 1A,B).
Day 4	- Diagnostic and therapeutic flexible bronchoscopy with BAL under non-intubated intravenous anesthesia.	- *Indications*: To confirm the suspected airway anomaly (tracheal bronchus). To perform therapeutic lavage for persistent left lower lobe atelectasis. - *Discovery*: Incidental confirmation of a displaced-type right tracheal bronchus (Figure 2).
Day 5	- Adjustment of antibiotic therapy based on BAL results - Clinical improvement - Antibiotic switched to (*Azithromycin*)	- *BAL result*: *Mycoplasma pneumoniae* detected by multiplex PCR. - Targeted therapy led to rapid resolution of symptoms
Follow-up	- Uneventful recovery and discharge - Family counseling regarding the tracheal bronchus	- To ensure complete resolution of infection and to inform the family of the incidental finding for future medical safety

BAL, bronchoalveolar lavage.

### 2.4 Anesthetic management and the incidental discovery

The bronchoscopy was performed under general anesthesia without endotracheal intubation. After securing intravenous access and instituting standard monitoring (electrocardiogram, non-invasive blood pressure, pulse oximetry), a carefully titrated regimen was employed: dexmedetomidine (0.2 µg/kg) and sufentanil (0.1 µg/kg) were administered as boluses for initial sedation and analgesia, followed by carefully titrated intermittent boluses of propofol (0.1–0.2 mg/kg) to achieve the desired depth of sedation while rigorously preserving spontaneous respiration and airway reflexes (13, 14). The patient's airway was maintained by a continuous jaw thrust maneuver by the attending anesthesiologist, with supplemental oxygen via a nasal cannula.

**Figure 2:**
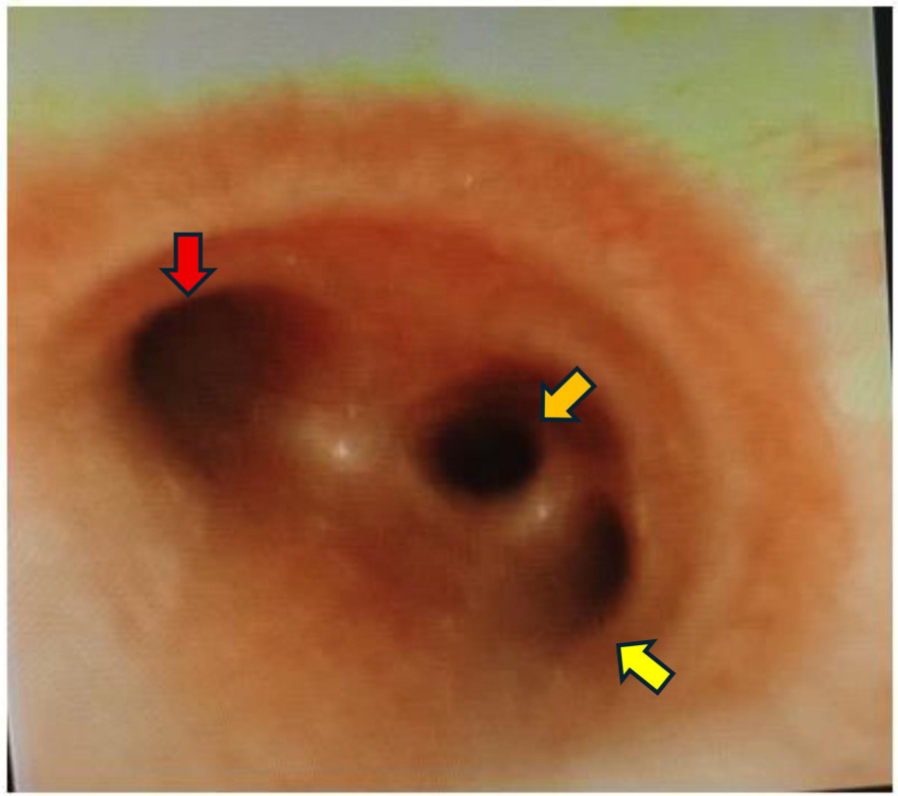
Bronchoscopic view of the tracheal bifurcation. Flexible bronchoscopy revealed the orifice of the tracheal bronchus (yellow arrow) located above the carina on the right lateral tracheal wall. The openings of the left main bronchus (red arrow) and the right main bronchus (orange arrow) were also visualized.

The bronchoscope was introduced into the airway through the nasal cavity by the attending pediatric pulmonologist. During advancement of the scope, the anesthesiology team, jointly observing the video feed, identified an anomalous orifice on the right lateral wall of the trachea, approximately 0.5 cm above the carina. A bronchoscopic examination confirmed the absence of the right upper lobe bronchus at its normal bifurcation from the right main bronchus, indicating that this was a “displaced”-type tracheal bronchus (where the entire right upper lobe bronchus originates from the trachea) rather than a “supernumerary” type (an accessory bronchus in addition to the normal branching pattern). Based on the modified Ghaye classification (10, 15, 16), this anomaly was characterized as a displaced-type tracheal bronchus supplying the apical segment of the right upper lobe (Figure 2). The bronchial mucosa of the TB appeared normal, without signs of inflammation, and was clearly unrelated to the left-sided pneumonic process. The bronchoalveolar lavage (BAL) was successfully performed in the affected left lower lobe.

### 2.5 Outcome and follow-up

A multiplex PCR assay performed on the BAL fluid confirmed *Mycoplasma pneumoniae* as the causative pathogen. The antibiotic therapy was adjusted accordingly, leading to a rapid clinical resolution. At a 2-week follow-up visit, the patient was asymptomatic, and a repeat chest X-ray showed complete resolution of the left lower lobe infiltration. The patient made an uneventful recovery and was discharged home. A formal consultation was held with the family to explain the incidental finding of the tracheal bronchus and its critical importance for any future anesthetic procedures. The patient's family expressed gratitude for the thorough explanation of the incidental finding and the guidance provided for future medical care, which alleviated their anxieties.

## 3 Discussion

This case serves as a paradigm for the extended diagnostic role of the anesthesiologist in perioperative medicine. The patient's acute pathology was definitively localized to the left lung and attributed to *Mycoplasma pneumoniae*. The right-sided TB was an entirely incidental discovery, a “silent” anomaly that would have otherwise remained undiagnosed. This aligns with the literature suggesting that a substantial number of tracheal bronchi are discovered incidentally and are not the cause of the presenting symptoms (7, 8). This fortuitous finding, however, carries immense significance for the patient's long-term safety. Knowledge of this anatomy is now pivotal; in any future scenario requiring airway instrumentation, strategies can be proactively adapted to avoid TB occlusion and its attendant complications (12, 16).

The successful and safe conduct of this bronchoscopy underscores the viability of a non-intubated, spontaneous ventilation approach for such diagnostic procedures in children. The chosen anesthetic technique was instrumental in this discovery, as it provided an optimal, unobstructed panoramic view of the native airway anatomy—a view that would have been obscured by an ETT placed in the high trachea, thereby potentially concealing the tracheal bronchus. The combination of dexmedetomidine, sufentanil, and propofol provided a stable platform of sedation and analgesia while maintaining respiratory drive, a technique well-suited for diagnostic bronchoscopy in pediatric patients (17). Recent evidence supports the safety and efficacy of such non-intubated, sedation-based techniques for pediatric airway endoscopy, emphasizing their value in preserving native airway dynamics for diagnostic accuracy (14). However, this method demands meticulous titration of sedatives and a high level of vigilance from the anesthesiologist to manage the airway without instrumentation. Therefore, the choice between intubated and non-intubated techniques should be individualized, weighing the need for an optimal anatomical view against the patient's specific clinical status and the team's expertise.

This report also clarifies that the presence of a TB does not invariably cause ipsilateral pulmonary pathology. The definitive identification of a contralateral infectious agent reinforces the principle that anatomical variants should not be presumed symptomatic without thorough investigation.

Our experience underscores the importance of including tracheobronchial anomalies in the differential diagnosis of a difficult airway. As highlighted in a recent narrative review on pediatric difficult airway management, proactive identification of congenital variants like the tracheal bronchus is a cornerstone of safe anesthetic practice, enabling the formulation of tailored airway strategies to prevent complications such as lobar collapse (18, 19).

Although this report is limited to a single case, it powerfully reiterates that the anesthesiologist's domain extends beyond physiological management to include anatomical diagnosis, with direct implications for a patient's lifelong medical safety.

## 4 Conclusion

We describe the unmasking of a silent tracheal bronchus during flexible bronchoscopy for contralateral pneumonia, a discovery made possible by the anesthetic approach employed. This case accentuates the anesthesiologist's crucial role as a perioperative physician whose observational skills can preempt future iatrogenic complications. It serves as a compelling reminder that in the practice of anesthesia, vigilance is essential to safeguarding airways, both in the immediate and in the distant future.

## Data Availability

The raw data supporting the conclusions of this article will be made available by the authors without undue reservation.
