# Supplementary material for: An incidental discovery of a silent tracheal bronchus during flexible bronchoscopy: a case report and anesthetic perspective
Source: Front Pediatr. 2026 Jan 6;13:1718566. doi: 10.3389/fped.2025.1718566 (PMC12816254; doi:10.3389/fped.2025.1718566)
Supplement: Supplementary file 1 [file Table1.docx]

**Table 1. Clinical Timeline and Management Decisions**

| Timepoint (Day) | Key Events & Interventions | Clinical Rationale & Findings |
| --- | --- | --- |
| **Day 1** | - Hospital admission.  - Prior oral amoxicillin-clavulanate for 3 days.  - Initiation of empiric intravenous antibiotics (*Ceftriaxone*) | - Persistent high fever and clinical signs of pneumonia necessitating inpatient care and IV therapy. |
| **Day 3** | - Persistent fever and lobar collapse on repeat chest X-ray.  - Performance of chest computed tomography (CT). | - Persistent fever after 48 hours of IV antibiotics.  - To evaluate the extent of consolidation, exclude complications (e.g., abscess, effusion), and investigate poor clinical response.  - **CT Finding:** Confirmed left lower lobe consolidation and **raised suspicion of a tracheal bronchus** (Figure 1A, B). |
| **Day 4** | - Diagnostic and therapeutic flexible bronchoscopy with BAL under non-intubated intravenous anesthesia. | - **Indications:**  1. To confirm the suspected airway anomaly (tracheal bronchus).  2. To perform therapeutic lavage for persistent left lower lobe atelectasis.  - **Discovery:** Incidental confirmation of a displaced-type right tracheal bronchus (Figure 2). |
| **Day 5** | - Adjustment of antibiotic therapy based on BAL results.  - Clinical improvement.  - Antibiotic switched to (*Azithromycin*) | - **BAL Result:** Mycoplasma pneumoniae detected by multiplex PCR.  - Targeted therapy led to rapid resolution of symptoms. |
| **Follow-up** | - Uneventful recovery and discharge.  - Family counseling regarding the tracheal bronchus. | - To ensure complete resolution of infection and to inform the family of the incidental finding for future medical safety. |

BAL: bronchoalveolar lavage
